# Diagnostic and prognostic value of echocardiography in pulmonary hypertension: an umbrella review of systematic reviews and meta-analyses

**DOI:** 10.1186/s12890-023-02552-y

**Published:** 2023-07-10

**Authors:** Tian-Xin Dong, Qing Zhu, Shi-Tong Wang, Yong-Huai Wang, Guang-Yuan Li, Fan-Xin Kong, Chun-Yan Ma

**Affiliations:** 1 Department of Cardiovascular Ultrasound, The First Hospital of China Medical University, No. 155 Nanjing Bei Street, Shenyang, 110001 Liaoning China; 2 Clinical Medical Research Center of Imaging in Liaoning Province, Shenyang, Liaoning China

**Keywords:** Echocardiography, Pulmonary hypertension, Diagnostic value, Right ventricular longitudinal strain, Umbrella review

## Abstract

**Background:**

The role of echocardiography in the diagnostic and prognostic assessment of pulmonary hypertension (PH) has been widely studied recently. However, these findings have not undergone normative evaluation and may provide confusing evidence for clinicians. To evaluate and summarize existing evidence, we performed an umbrella review.

**Methods:**

Systematic reviews and meta-analyses were searched in PubMed, Embase, Web of Science, and Cochrane Library from inception to September 4, 2022. The methodological quality of the included studies was assessed using Assessment of Multiple Systematic Reviews (AMSTAR), and the Grading of Recommendations Assessment, Development and Evaluation (GRADE) system was used to evaluate the quality of evidence.

**Results:**

Thirteen meta-analyses (nine diagnostic and four prognostic studies) were included after searching four databases. The methodological quality of the included studies was rated as high (62%) or moderate (38%) by AMSTAR. The thirteen included meta-analyses involved a total of 28 outcome measures. The quality of evidence for these outcomes were high (7%), moderate (29%), low (39%), and very low (25%) using GRADE methodology. In the detection of PH, the sensitivity of systolic pulmonary arterial pressure is 0.85–0.88, and the sensitivity and specificity of right ventricular outflow tract acceleration time are 0.84. Pericardial effusion, right atrial area, and tricuspid annulus systolic displacement provide prognostic value in patients with pulmonary arterial hypertension with hazard ratios between 1.45 and 1.70. Meanwhile, right ventricular longitudinal strain has independent prognostic value in patients with PH, with a hazard ratio of 2.96–3.67.

**Conclusion:**

The umbrella review recommends echocardiography for PH detection and prognosis. Systolic pulmonary arterial pressure and right ventricular outflow tract acceleration time can be utilized for detection, while several factors including pericardial effusion, right atrial area, tricuspid annular systolic displacement, and right ventricular longitudinal strain have demonstrated prognostic significance.

**Trial registration:**

PROSPERO (CRD42022356091), https://www.crd.york.ac.uk/prospero/.

**Supplementary Information:**

The online version contains supplementary material available at 10.1186/s12890-023-02552-y.

## Background

Pulmonary hypertension (PH) is a multi-etiological condition characterized by abnormally increased pulmonary artery pressure [1, 2]. The 2022 ESC/ERC guidelines defined PH as a mean pulmonary artery pressure (mPAP) > 20 mmHg measured by right heart catheterization (RHC) at rest [3]. PH mainly manifests as progressive right cardiac dysfunction and is associated with high morbidity, mortality, and various complications [1–4]. Therefore, timely diagnosis, evaluation, and treatment are particularly important in patients with PH.

The gold standard for diagnosing PH is invasive RHC, [3] which is difficult to perform widely in clinical practice. Echocardiography is widely used in the preliminary screening of PH because it is non-invasive, convenient, and accurate. Echocardiography mainly estimates the probability of PH by estimating systolic pulmonary arterial pressure (sPAP) and combining other manifestations; however, the role of echocardiography in the diagnosis of PH remains controversial [3–5]. Furthermore, guidelines recommend the use of right atrial area (RAA), tricuspid annular systolic displacement (TAPSE)/sPAP and pericardial effusion (PE) to evaluate right ventricular function in patients with PH and predict prognosis [3]. However, the prognostic value of these parameters remains unclear. Thus, recently, several systematic reviews and meta-analyses have been conducted on the role of echocardiography in the diagnosis and prognosis of PH [6–18]. Nevertheless, methodological pitfalls, inconsistency between original studies, and the risk of bias lead to inaccurate results. Therefore, we need a method to systematically and comprehensively evaluate the quality of the existing evidence.

Umbrella reviews, also called overviews of systematic reviews, help to analyze, evaluate, and summarize systematic reviews of a specific topic and improve the authenticity and reliability of evidence [19]. Therefore, we conducted this umbrella review to evaluate the ability of echocardiography in the diagnosis and prognosis of PH, aiming to provide an accurate and comprehensive reference for clinicians.

## Methods

### Protocol registration and literature search

The umbrella review was performed adhering to the Preferred Reporting Items for Systematic Reviews and Meta-Analyses (PRISMA) guidelines (Supplementary files) and prospectively registered with PROSPERO (No: CRD42022356091) [20]. PubMed, Embase, Web of Science, and Cochrane Library databases were systematically searched with the keywords “pulmonary hypertension,” “systematic review and meta-analysis,” and “echocardiography” to identify relevant studies from inception until September 4, 2022. The detailed search strategy is shown in S1. To avoid missing relevant studies, the references of the included studies were hand-searched.

### Inclusion and exclusion criteria

The inclusion criteria were as follows:i) systematic review or meta-analyses published in English; ii) echocardiography was used to evaluate the diagnostic or prognostic values of PH;iii) the key data was reported (sensitivity, specificity, area under the curve, and hazard ratio [HR]); and iv) RHC as the gold standard for the diagnosis of PH. The exclusion criteria were as follows:i) key data not reported, or inability to obtain data from the meta-analyses;ii) the number of included studies was less than 3;iii) narrative reviews, commentaries, conference abstracts, etc.; and iv) studies not published in English.

### Article selection and data extraction

Two authors (Tianxin Dong and Qing Zhu) independently screened the articles according to predefined inclusion and exclusion criteria, and the disagreement was resolved by a third person (Shitong Wang). The following data were independently extracted by two authors:i) study information: first author, year of publication, country, the number of studies included in the meta-analysis, and the quality evaluation tool;ii) echocardiographic parameters;iii) critical data (sensitivity, specificity, area under the curve, and hazard ratio); and iv) main conclusions. All information was checked by a third person and summarized in Tables 1 and 2.

**Table 1:** Main characteristics and evidence quality of diagnostic meta-analyses

References	Country	No. of Studies	Quality Assessment	Measurement Parameters	AUC	Outcome	Downgrade Factors					Certainty of the evidence
							**Risk of bias**	**Inconsistency**	**Indirectness**	**Imprecision**	**Publication bias**	
Zhang,2010	China	6	QUADAS & STARD	sPAP/RVSP	0.82	SE, 0.82 SP, 0.68	0 0	-2^b^ -2^b^	0 0	-1^e^ -1^e^	0 0	very low very low
Janda,2011	Canada	29^ g^	QUADAS	RVSP	0.86	SE, 0.83 SP, 0.72 *R*^h^, 0.70	-1^a^ -1^a^ -1^a^	-1^c^ -1^c^ -2^b^	0 0 0	-^1e^ -1^e^ -1^e^	0 0 -1^f^	very low very low very low
Taleb,2013	America	9	NR	sPAP	NR	SE, 0.88 SP, 0.63	-1^a^ -1^a^	0 -1^d^	0 0	0 0	0 0	moderate low
Wang,2018	China	21	QUADAS	RVOT AT	0.90	SE, 0.84 SP, 0.84	0 0	-1^c^ -1^d^	0 0	0 0	0 0	moderate moderate
Ni,2019	China	27	QUADAS-2	sPAP/TRPG	0.88	SE, 0.85 SP, 0.74	0 0	-1^c^ -1^c^	0 0	-1^e^ -1^e^	0 0	low low
Ullah,2020	America	27	QUADAS-2	sPAP	NR	SE, 0.85 SP, 0.71	-1^a^ -1^a^	-1^c^ -1^c^	0 0	0 0	-1^f^ -1^f^	very low very low
Tsujimoto,2022	Japan	17	QUADAS-2	RAP	NR	SE, 0.87 SP, 0.86	-1^a^ -1^a^	-1^c^ -1^c^	0 0	0 0	0 0	low low
Korbitz,2020	America	13	QUADAS-2	sPAP	0.81	SE, 0.86 SP, 0.87	0 0	0 -2^b^	0 0	0 0	0 0	high low
Yin,2020	China	11	QUADAS-2	sPAP	0.91	SE, 0.85 SP, 0.83	0 0	-1 -2^b^	0 0	0 0	0 0	moderate low

*sPAP* systolic pulmonary arterial pressure, *RVSP* right ventricular systolic pressure, *RVOT AT* right ventricular outflow tract acceleration time, *TRPG* tricuspid regurgitation pressure gradient, *RAP* right atrial pressure, *NR* not reported, *AUC* area under the curve, *SE* sensitivity, *SP* specificity, *QUADAS* quality assessment of diagnostic studies tool

^a^quality assessment suggested risk of bias

^b^high heterogeneity (I^2^ > 75%)

^c^high heterogeneity (I^2^ > 75%), but heterogeneity was mainly explained

^d^existence of heterogeneity (50% < I^2^ ≤ 75%); e.PH diagnosed by different methods or threshold

^f^asymmetry on funnel plot

^g^Of the 29 studies, 27 studies performed meta-analysis of correlation coefficients and 12 studies performed meta-analysis of diagnostic accuracy

^h^correlation coefficients between RVSP and right heart catheterization-derived sPAP

**Table 2:** Main characteristics and evidence quality of prognostic meta-analyses

References	Country	No. of studies	Measurement Parameters	Outcomes	Effect Size	Downgrade Factors					Upgrade Factors	Certainty of the evidence
						**Risk of bias**	**Inconsistency**	**Indirectness**	**Imprecision**	**Publication bias**		
Baggen,2016	Holland	27	PE RAA TAPSE	endpoint event ^a^ endpoint event ^a^ endpoint event ^a^	HR, 1.70 HR, 1.71 HR, 1.72	0 0 0	0 -1^d^ -1^e^	0 0 0	-1^f^ 0 0	0 -1^ h^ -1^ h^	no no no	moderate low low
Shukla,2018	Canada	10	RVLS TAPSE	all-cause mortality all-cause mortality	HR, 3.67 HR, 1.45	-1^c^ -1^c^	0 0	0 0	-1^ g^ 0	0 0	large effect ^j^ no	moderate moderate
Hulshof,2018	Holland	11	RVLS RVLS	all-cause mortality combined endpoint ^b^	HR, 2.96 HR, 1.22	0 0	0 -1^d^	0 0	0 0	-1^i^ -1^i^	large effect^j^ no	high low
Liu,2020	China	12	RAA/RAAI RAA/RAAI	all-cause mortality combined endpoint ^b^	HR, 1.50 HR, 1.53	0 0	0 -1^e^	0 0	-1^f^ -1^f^	0 0	no no	moderate low

*PE* pericardial effusion, *RAA* right atrial area, *RAAI* right atrial area index, *TAPSE* tricuspid annular plane systolic excursion, *RVLS* right ventricular longitudinal strain, *HR* hazard ratio, *NR* not reported

^a^defined as death, transplantation or clinical deterioration

^b^defined as death or PH related events

^c^quality assessment suggested risk of bias

^d^existence of heterogeneity (50% < *I*^*2*^ ≤ 75%)

^e^high heterogeneity (*I*^*2*^ > 75%), but heterogeneity was mainly explained

^f^different methods of prognosis

^g^RV strain included free wall, septum, and global strain

^h^asymmetry on funnel plot; i. publication bias was not assessed; j. HR > 2

### Methodological quality assessment

The methodological quality of the included systematic reviews and meta-analyses was assessed using Assessment of Multiple Systematic Reviews (AMSTAR) [21]. The AMSTAR contains 11 checklists, and each item requires a yes, no, unclear, or unused answer. One point for each yes response and the cumulative scores of the 11 items classified studies into high (8–11 points), moderate (4–7 points), and low (0–3 points) quality. Scoring was independently conducted by two people (Tianxin Dong and Qing Zhu) and checked by a third person (Shitong Wang), and disagreements were addressed by discussion.

### Evaluation of degree of overlap

The overlap of the original study was evaluated using the overall corrected covered area (CCA) and the pairwise CCA [22]. Overall CCA assesses the total overlap, [23] while pairwise CCA examines the overlap between each pair of meta-analyses included in the study [24]. The CCA range 0% to 5% indicates slight overlap, 6% to 10% indicates moderate overlap, 11% to 15% indicates high overlap, and above 15% indicates very high overlap. This work was conducted by Tianxin Dong and Qing Zhu, with verification by Chunyan Ma.

### Grading of the evidence

The Grading of Recommendations Assessment, Development and Evaluation (GRADE) system was used to evaluate the quality of evidence. The GRADE system includes five downgrade factors (risk of bias, inconsistency, indirectness, imprecision, and publication bias) and three upgrade factors (large effect value, dose–effect relationship, and a possible confounding bias that may reduce efficacy) and divides the evidence into four grades: high, moderate, low, and very low [25, 26]. High and moderate evidence is used to recommend echocardiographic parameters, while low and very low evidence is used to recommend against the use of a parameter [26]. For the studies based on diagnostic test accuracy and prognosis, the initial quality of evidence was high [27]. This work was independently conducted by two authors (Tianxin Dong and Qing Zhu) and reviewed by a third author (Chunyan Ma), and any discrepancies were resolved by discussion.

## Results

### Literature search and selection

**Fig. 1:**
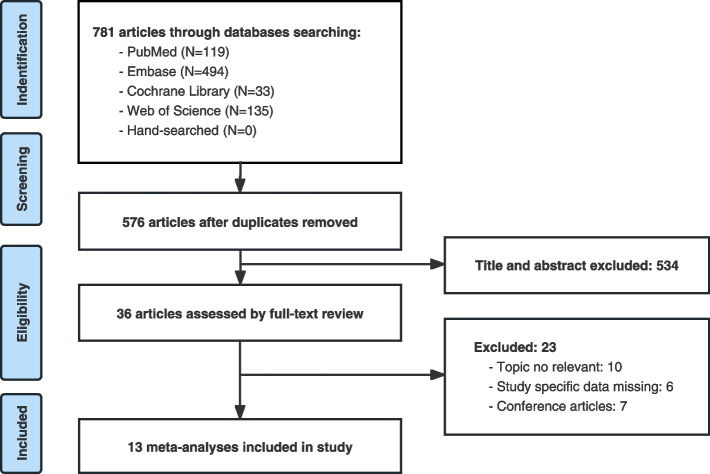
Flowchart of the study selection process

Based on the pre-formulated search strategy, 781 articles were retrieved from the four databases. After eliminating duplicates (*n* = 205), the remaining 576 articles were screened based on their titles and abstracts (*n* = 540). 36 articles that met the inclusion criteria were screened by full-text reading, and 23 articles were excluded. Ultimately, 13 articles were included in the umbrella review [6–18]. No additional studies were retrieved from manually searching the references of the included articles. The flowchart in Fig. 1 shows the detailed process and reasons for exclusion.

### Study characteristics of diagnostic meta-analysis

Nine meta-analyses published between 2010 and 2022 reported the sensitivity and specificity of echocardiography in the detection of PH, and the number of included studies varied between 6 and 29 (Table 1) [6–8, 11, 13, 15–18]. Seven meta-analyses did not have specific constraints on the etiology of PH, whereas two meta-analyses included patients with portopulmonary hypertension (POPH) [15, 16]. The echocardiographic methods used to identify probable PH include sPAP, right ventricular systolic pressure (RVSP), right atrial pressure (RAP), tricuspid regurgitation pressure gradient (TRPG), and right ventricular outflow tract acceleration time (RVOT AT). The sensitivity ranged from 0.82 to 0.88, the specificity ranged from 0.63 to 0.87, and the area under the curve (AUC) ranged from 0.81 to 0.91. The AUC of sPAP for detecting POPH was the highest (0.91) [16], and the AUC of RVOT AT was also high (0.90) for detecting PH among these parameters [11]. Moreover, eight of the nine meta-analyses used quality assessment of diagnostic studies tool (QUADAS) or QUADAS-2 for quality assessment, whereas one meta-analysis did not report quality assessment tools [8].

### Study characteristics of prognostic meta-analysis

Four meta-analyses have reported the prognostic value of echocardiography in patients with PH (Table 2) [9, 10, 12, 14]. The studies were published between 2016 and 2020, and the number of original studies included ranged from 10 to 27. A total of 5 parameters were evaluated: PE, RAA, right atrial area index (RAAI), TAPSE, and right ventricular longitudinal strain (RVLS). Except for RVLS, the remaining parameters are limited to patients with pulmonary arterial hypertension (PAH) rather than all PH patients. The main outcome indicators included endpoint event, all-cause mortality, and combined endpoint. The specific definitions can be found in Table 2. The HR was between 1.22 and 3.67 for the above-mentioned parameters. Additionally, each of the four meta-analyses used the quality assessment tool for prognostic studies proposed by Hayden et al. [28].

### Methodological quality of the included studies

The results of AMSTAR showed (S2) that of the 13 included meta-analyses, 8 studies were of high quality (62%), 5 were of moderate quality (38%), and no low-quality studies were identified. The main problems were that the list of studies (included and excluded) was not provided (85%), restrictions of publication language and study type were not clearly described (77%), no design scheme was provided (69%), and the literature search was not comprehensive (38%). In addition, publication bias was not assessed in the two meta-analyses.

### Degree of overlap

The degree of overlap was presented in Supplementary Fig. 1. In the case of nine diagnostic meta-analyses, CCA amounted to 8.06%, which indicates moderate overlapping. A total of 36 nodes were formed, with pairwise CCA indicating that 19% of the nodes had very high overlap. The Yin and Korbitz's studies had the highest overlap, which explained that the sensitivity of sPAP in diagnosing PH in patients with POPH was greatly influenced by overlap [15, 16]. Regarding the four prognostic meta-analyses, the overall CCA was 7.14%, indicating a moderate overlap. Six nodes were formed, and the overlap between Shukla and Hulshof's studies was very high, [10, 12] which revealed that the results of RVLS obtained from these two studies were significantly influenced by the overlapping studies, indicating a certain degree of bias.

### Grading of the evidence

The 13 meta-analyses included 28 outcome measures. A total of 10 pieces of evidence were recommended, from 4 diagnostic and 4 prognostic meta-analyses [9, 10, 12, 14–16, 29]. The results of GRADE (Table 1 and 2) showed that two pieces of evidence were supported by high evidence (7%), and eight pieces of evidence were supported by moderate evidence (29%). In addition, 11 pieces of evidence presented low evidence (39%), and the other seven were supported by very low evidence (25%). The main downgrading factors were inconsistency (75%), risk of bias (39%), imprecision (39%) and publication bias (25%). Two outcome measures were upgraded owing to large effect sizes.

## Discussion

### Principal findings

We performed this umbrella review according to the results of AMSTAR and the grading of the evidence, which indicated good performance of echocardiography for the tentative diagnosis and prognostication of PH. For the detection of PH, sPAP had high sensitivity but relatively low specificity, and RVOT AT had both high sensitivity and specificity. For the prognosis prediction, PE, RAA and TAPSE provide prognostic value in patients with PAH. RVLS has independent prognostic value in patients with PH.

### Echocardiography for detecting PH

RHC, which can directly measure pulmonary artery pressure, is considered the gold standard method to diagnose PH [3]. However, it is invasive, costly, and has potentially fatal complications; therefore, it cannot be widely performed. In view of this, echocardiography is an effective tool for detecting PH, as it is non-radiating, non-invasive, and accurate. sPAP estimated by the tricuspid regurgitation method was the most commonly used method, [30] and some studies used RAP, RVSP, etc., instead of sPAP to detect PH [6, 7, 13]. Recently, RVOT AT has been used to detect PH, but it has not yet been widely deployed [11]. It should be noted that echocardiography is helpful for screening, but insufficient for diagnosis at present, and the accuracy of echocardiography in the diagnosis of PH is often questioned. Therefore, several meta-analyses have been conducted to evaluate the diagnostic efficacy of echocardiography, which must be evaluated and pooled. Our umbrella review recommended four meta-analyses that involved one piece of high evidence and four pieces of moderate evidence [8, 11, 15, 16].

Three of the four recommended meta-analyses evaluated the value of sPAP in diagnosing PH [8, 15, 16]. All included studies used the tricuspid regurgitation velocity (TRV) and estimated RAP to estimate sPAP. RAP estimation was mainly calculated through the diameter and collapse rate of the inferior vena cava during natural respiration. However, some studies calculated it through jugular vein pressure, which may be one of the reasons for the high heterogeneity of the results. Taleb et al. demonstrated that the sensitivity of sPAP in the diagnosis of PH was 0.88 [8]. Two meta-analyses showed that, in patients with POPH, the sensitivity of sPAP was 0.86, and the AUC was 0.91 [15, 16]. Meanwhile, it is suggested to set the diagnostic threshold of sPAP for detecting POPH as 40–45, with a sensitivity of 0.86 and specificity of 0.87 [15]. A study by Ni et al. showed that the sensitivity of sPAP in the detection of patients with PH and pulmonary disease is significantly decreased, and it is not recommended to use echocardiography to measure sPAP in such patients [13]. The present evidence indicates that sPAP exhibits high sensitivity in detecting PH, thereby playing a crucial role in screening PH. However, it is worth noting that guidelines suggest using TRV to detect PH due to the imprecision in RAP estimation and errors caused by derived variables [3]. Since there is currently no meta-analysis evaluating TRV's precision in detecting PH, we did not feature it as the primary recommended parameter. Thus, further investigation is necessary to clarify the accuracy of TRV in detecting PH.

A meta-analysis involving 21 original studies and 1280 patients with PH evaluated the value of RVOT AT in the diagnosis of PH [11]. RVOT AT refers to the time interval from the beginning of right ventricular ejection to peak flow velocity across the pulmonary valve, which is strongly correlated with sPAP (*r* = -0.83) and can be measured in most patients [31]. This study showed that RVOT AT had high sensitivity and specificity, with an AUC of 0.9, regardless of the etiology of PH. For patients with arrhythmias, the specificity of RVOT AT was not affected and its sensitivity increased to 0.94 [11]. RVOT AT provides a new method for the detection of PH by echocardiography, especially for patients whose TRPG cannot be measured, and it is expected to be used as a supplementary method to enhance the diagnostic accuracy of echocardiography for PH. But it should be noted that the different diagnostic thresholds and the different methods of transthoracic or transesophageal echocardiography have a great impact on the sensitivity of the diagnosis. The higher the diagnostic threshold, the higher the sensitivity. The sensitivity of measuring RVOT AT by transthoracic echocardiography is higher than that of transesophageal echocardiography. The latest guidelines also set 105 ms as the cut-off value for RVOT AT, [3] and this recommendation needs to be further verified by more large-sample, high-quality, and multicenter studies.

In addition, some meta-analyses have evaluated the value of RVSP and RAP in the diagnosis of PH [6, 7, 18]. Although they reported high sensitivity and specificity, the inconsistency, imprecision, and risk of bias of the results resulted in a low or very low quality of evidence and should not be recommended. More high-quality studies are required to verify these results.

In summary, the sensitivity of sPAP in detecting PH is high while the specificity is relatively low, and RVOT AT has high sensitivity and specificity in the detection of PH. Although sPAP and RVOT AT are effective in detecting PH, RHC remains crucial for accurate diagnosis. Additionally, there is hope that combining multiple parameters will enhance the diagnostic value of echocardiography for PH.

### Echocardiography prognosis values

Despite recent improvements in diagnosis and treatment, PH is still associated with a high mortality rate. The survival rate of patients with PH decreased year-by-year and was approximately 50% at seven years after the initial diagnosis [1]. Therefore, an accurate and effective prognostic evaluation of patients with PH is clinically important. There are currently several approaches to evaluate the prognosis of PH, such as the six-minute walking test, cardiopulmonary exercise test, and hemodynamic parameters measured by RHC, [3] and some risk scores have been proposed, such as REVEAL 2.0, COMPERA, and FPHR [29, 32]. Although these methods and risk scores are useful in predicting survival, echocardiography also plays an important role in prognosis because it is quantitative and allows the evaluation of right heart function and changes in pulmonary artery pressure. Various echocardiographic parameters, such as RAA, TAPSE/sPAP, PE, and RV strain can reflect the severity and prognosis of PH, [3] and many meta-analyses have explored the prognostic value of different parameters. One piece of high evidence and four pieces of moderate evidence were recommended by our umbrella review [9, 10, 12, 14].

Currently, right ventricular ejection fraction measured by three-dimensional echocardiography, RV-FAC, TAPSE, and tricuspid annular systolic velocity (s′) are commonly used in clinics to assess RV systolic function [3, 30]. Recently, the role of RV strain measured by speckle tracking echocardiography has increasingly recognized and emphasized in the early evaluation of RV systolic function [33]. Two meta-analyses used RVLS to judge the prognosis of patients with PH [10, 12]. Shukla et al. showed that the HR of RVLS for predicting all-cause mortality in PH was 3.67, which was better than that of TAPSE [10]. A previous study showed that patients with PH with a 22% relative reduction in RVLS had a significantly higher risk of all-cause mortality (HR = 2.96) [12]. It is worth mentioning that in patients with PH and Eisenmenger syndrome, RV transverse strain had a higher predictive value than RVLS, [34] which suggests that RV strain in other directions may have equal prominence to RVLS and should not be ignored in future studies.

PE, which is closely related to prognosis, is a common clinical presentation of PAH [35]. Baggen et al. showed that PE was an independent predictor of death, transplantation, and clinical deterioration in patients with PAH, with an HR of 1.70 [9]. The right atrium is important for blood storage, and PAH can elevate RAP and result in enlargement of the right atrium. A study suggested that RAA/RAAI was associated with an increased risk of poor prognosis in patients with PAH (HR = 1.50) [14]. TAPSE is a convenient indicator of right ventricular longitudinal systolic function and prognosis, [36] and the HR of TAPSE for all-cause mortality in patients with PAH was 1.45, [10] which could be used as an independent prognostic factor with a limited predictive value.

The TAPSE/sPAP ratio is a crucial parameter for non-invasive assessment of RV-arterial coupling. It offers a valuable alternative to measuring the end-systolic/arterial elasticity ratio via invasive pressure–volume loops. TAPSE/sPAP can enlighten clinicians on the diastolic stiffness of the right ventricle in patients suffering from severe PH, and play a key role in prognosis [37, 38]. The 2022 ESC guidelines have also recognized it as an important prognostic factor, with thresholds of 0.32 and 0.19 for high, medium, and low risk [3]. Nevertheless, there has been no systematic review or meta-analysis on this parameter to allow evidence to be evaluated by GRADE. With an increasing number of studies, a future meta-analysis on the prognostic value of TAPSE/sPAP ratio may provide more dependable clinical evidence.

Therefore, PE, RAA, and TAPSE are prognostic factors for PAH. RVLS is recommended as a critical prognostic tool because it is an independent prognostic factors and has high HR, and we are confident that RVLS will provide incremental prognostic value to echocardiography or risk stratification models in the future.

### Study limitations

This umbrella review has several limitations. First, the quality of the umbrella review depends on the quality of the included meta-analyses. Different etiologies and inconsistent diagnostic cut-off values may affect the strength and reliability of evidence in meta-analyses. Second, in the original studies, all the patients with PH included in the study groups were confirmed by RHC. These patients were initially suspected of PH based on clinical indications and echocardiography. As a result, the sensitivity and specificity of echocardiography in our study may have been inflated. Therefore, the results of our study should be interpreted with caution when extrapolating to non-high-risk populations. Third, some patients with PH may have been excluded because RHC was not required for those whose echocardiography was “normal”, which resulted in a population selection bias. And the control groups in some of the included studies did not undergo RHC to confirm the absence of PH, which may lead to the inclusion of some patients with PH.

Fourth, the population heterogeneity is an issue that cannot be ignored in this meta-analysis on the topic. PH involves many disease etiologies and many patients with PH also have comorbidities such as heart and lung diseases, [3] which can affect the results of echocardiography measurements. For example, different types of heart failure have different effects on the right heart, which in turn affects parameters such as sPAP; some connective tissue diseases also have different effects on cardiac function [18]. In addition, differences in race in original studies can also lead to differences in results.

Fifth, although we evaluated the overlap of original studies, there is currently no clear method to assess the specific impact of overlap. Next, only studies published in English were included; therefore, the risk of language bias could not be ignored. Finally, the evaluation of the AMSTAR and GRADE systems were affected by subjective factors, even though they were performed independently by two authors and checked by a third person.

### Future research

According to the umbrella review, some recommendations and ideas for future research include: i) future studies are needed to categorize the etiology of PH to avoid the influence of primary diseases on the results; ii) the time interval between RHC and echocardiography should be controlled and shortened as much as possible because it is an important factor that affects the accuracy of echocardiography diagnosis;iii) future research should clarify that echocardiography can only be used for the detection of PH at present, not diagnosis;iv) multi-parameter models can be constructed to further improve the diagnostic and prognostic value of echocardiography for PH;v) it is important to investigate the prognostic value of PE, RAA, and TAPSE in other types of PH since they are currently limited to PAH patients. Additionally, the sample size of RVLS is still small, and further research is needed to expand its applicability; and vi) further clarification is needed on the impact of different degrees of PH severity on the accuracy of echocardiographic diagnosis.

It should be emphasized that the guidelines redefined the diagnostic criteria for PH (mPAP > 20 mmHg), whereas existing studies have used the old cut-off (25 mmHg). The downshift of diagnostic criteria is necessary for early diagnosis and treatment of PH; however, there are also effects on the reliability of the existing evidence. Therefore, further studies are urgently required to validate the applicability and authenticity of this evidence.

In future meta-analyses, the following issues should be noted:i) gray literature is an important source of information that should be considered when conducting the literature search;ii) 85% of the included meta-analyses did not provide a list of excluded articles, which we recommend to increase the credibility of meta-analyses in future; iii) 75% of the outcome measures were downgraded due to heterogeneity, which was one of the most important factors affecting the quality of evidence. Subgroup, meta-regression, and sensitivity analyses should be used to address heterogeneity. If heterogeneity cannot be explained, quantitative analysis is not recommended, and iv) 39% of the evidence is at risk of bias. An appropriate risk of bias tool should be selected according to the study design included in the meta-analysis, and studies with a high risk of bias should be carefully considered for inclusion.

## Conclusions

According to the findings of this umbrella review, echocardiography can be clinically used for the tentative diagnosis and prognosis of PH. The sensitivity of sPAP in detecting PH is high while the specificity is relatively low, thus it can be used for preliminary screening of PH. RVOT AT has high sensitivity and specificity in the detection of PH. Nevertheless, RHC remains necessary for accurate confirmation of the diagnosis. For the prognosis prediction, PE, RAA, and TAPSE provide prognostic value in patients with PAH. RVLS has independent prognostic value in patients with PH.

In future, it is important to use multiple echocardiographic parameters for detection and predicting the prognosis of PH. Therefore, multi-parameter models should be constructed to increase the diagnostic and prognostic value of echocardiography in PH. Furthermore, because the diagnostic criteria for PH have changed, further studies are urgently needed to verify the applicability of the findings obtained.

## Supplementary Information

**Additional file 1: Supplementary Table 1.** Detailed search strategy. **Supplementary Table 2.** Detailed of results for AMSTAR. **Supplementary Figure 1.** Overall overlap assessment and pairwise overlap assessment.

## Data Availability

All data generated or analyzed in the course of this study are included in these published articles and their supplementary information files, as detailed in the Supplementary Materials.

## Abbreviations

AMSTAR: Assessment of Multiple Systematic Reviews
AUC: Area under the curve
CCA: Corrected covered area
GRADE: Grading of Recommendations Assessment, Development and Evaluation
PE: Pericardial effusion
PH: Pulmonary hypertension
mPAP: Mean pulmonary artery pressure
RAA: Right atrial area
RAAI: Right atrial area index
RAP: Right atrial pressure
RHC: Right heart catheterization
RV-FAC: Right ventricular fractional area change
RVLS: Right ventricular longitudinal strain
RVOT AT: Right ventricular outflow tract acceleration time
RVSP: Right ventricular systolic pressure
sPAP: Systolic pulmonary arterial pressure
TAPSE: Tricuspid annulus systolic displacement
TRPG: Tricuspid regurgitation pressure gradient
TRV: Tricuspid regurgitation velocity
