# Supplementary material for: Diagnostic and prognostic value of echocardiography in pulmonary hypertension: an umbrella review of systematic reviews and meta-analyses
Source: BMC Pulm Med. 2023 Jul 10;23:253. doi: 10.1186/s12890-023-02552-y (PMC10334642; doi:10.1186/s12890-023-02552-y)
Supplement: Supplementary file 1 — Additional file 1: Supplementary Table 1. Detailed search strategy. Supplementary Table 2. Detailed of results for AMSTAR. Supplementary Figure 1. Overall overlap assessment and pairwise overlap assessment. [file 12890_2023_2552_MOESM1_ESM.docx]

**Supplemental Material**

**Supplementary Table 1.** Detailed search strategy

**Supplementary Table 2.** Detailed of results for AMSTAR

**Supplementary Figure 1.** Overall overlap assessment and pairwise overlap assessment

**Availability of data and materials**

**PRISMA checklist**

**Supplementary Table 1.** Detailed search strategy

| **Database** | **Search Strategy (September 4, 2022)** | **N (781)** |
| --- | --- | --- |
| **PubMed** | (("echocardiography"[All fields] OR "echocardiogram"[All fields] OR "echocardiographic"[All fields] OR "ultrasound"[All fields] OR "TTE"[ALL fields] OR "TEE"[All fields] OR "3DE"[All fields] OR "2DE"[All fields]) AND ("pulmonary hypertension"[All Fields] OR "pulmonary arterial hypertension"[All Fields] OR "PH"[All Fields] OR "PAH"[All Fields])) AND ("systematic review"[Title/Abstract] OR "meta"[Title/Abstract] OR "meta-analysis"[Title/Abstract]) | 119 |
| **Embase** | #1 ph OR 'pulmonary hypertension' OR 'pulmonary arterial hypertension' OR pah  #2 'systematic review' OR meta OR 'meta-analysis'  #3 echocardiography OR tee OR 2de OR tte OR 3de OR echocardiogram OR echocardiographic OR ultrasound  #1 AND #2 AND #3 AND [english]/lim | 494 |
| **Cochrane Library** | #1 ("pulmonary hypertension") or ("pulmonary arterial hypertension")  #2 echocardiography  #3 ("systematic review") or (meta) OR ("meta-analysis")  #4 #1 and #2 and #3 | 33 |
| **Web of Science** | 1: (TI=(meta-analysis)) OR TI=(systematic review)  2: (((((((TS=(echocardiography)) OR TS=(echocardiogram)) OR TS=(echocardiographic)) OR TS=(ultrasound)) OR TS=(TEE)) OR TS=(TTE)) OR TS=(3DE)) OR TS=(2DE)  3: (((TS=(pulmonary hypertension)) OR TS=(pulmonary arterial hypertension)) OR TS=(PH)) OR TS=(PAH)  4: #3 AND #2 AND #1 | 135 |

**Supplementary Table 2.** Detailed of results for AMSTAR

| **References** | **1** | **2** | **3** | **4** | **5** | **6** | **7** | **8** | **9** | **10** | **11** | **Score** | **Quality** |
| --- | --- | --- | --- | --- | --- | --- | --- | --- | --- | --- | --- | --- | --- |
| Zhang,2010 | N | Y | Y | U | N | Y | Y | Y | Y | Y | Y | 8 | H |
| Janda,2011 | N | Y | Y | Y | N | Y | Y | Y | Y | Y | Y | 9 | H |
| Taleb,2013 | N | Y | U | U | N | Y | Y | Y | Y | Y | Y | 7 | M |
| Wang,2018 | N | Y | Y | N | N | Y | Y | Y | Y | Y | Y | 8 | H |
| Ni,2019 | Y | Y | Y | N | N | Y | Y | Y | Y | Y | Y | 9 | H |
| Ullah,2020 | N | Y | U | N | N | Y | U | Y | Y | N | Y | 5 | M |
| Tsujimoto,2022 | Y | Y | Y | Y | Y | Y | Y | Y | Y | Y | Y | 11 | H |
| Korbitz,2020 | N | Y | U | N | N | Y | Y | Y | Y | Y | Y | 7 | M |
| Yin,2020 | Y | Y | Y | N | N | Y | Y | Y | Y | Y | Y | 9 | H |
| Baggen,2016 | Y | Y | U | Y | N | Y | Y | Y | Y | Y | Y | 9 | H |
| Shukla,2018 | N | Y | Y | N | Y | Y | Y | Y | Y | Y | Y | 9 | H |
| Hulshof,2018 | N | Y | Y | N | N | Y | Y | Y | Y | N | Y | 7 | M |
| Liu,2020 | N | Y | U | N | N | Y | Y | Y | Y | Y | Y | 7 | M |

Y: Yes; U: Unclear; N: No; H: High; M: Moderate. Items: 1. Was an 'a priori' design provided？2. Was there duplicate study selection and data extraction? 3. Was a comprehensive literature search performed? 4. Was the status of publication (i.e. grey literature) used as an inclusion criterion? 5. Was a list of studies (included and excluded) provided? 6. Were the characteristics of the included studies provided? 7. Was the scientific quality of the included studies assessed and documented? 8. Was the scientific quality of the included studies used appropriately in formulating conclusions? 9. Were the methods used to combine the findings of studies appropriate? 10. Was the likelihood of publication bias assessed? 11. Was the conflict of interest stated?


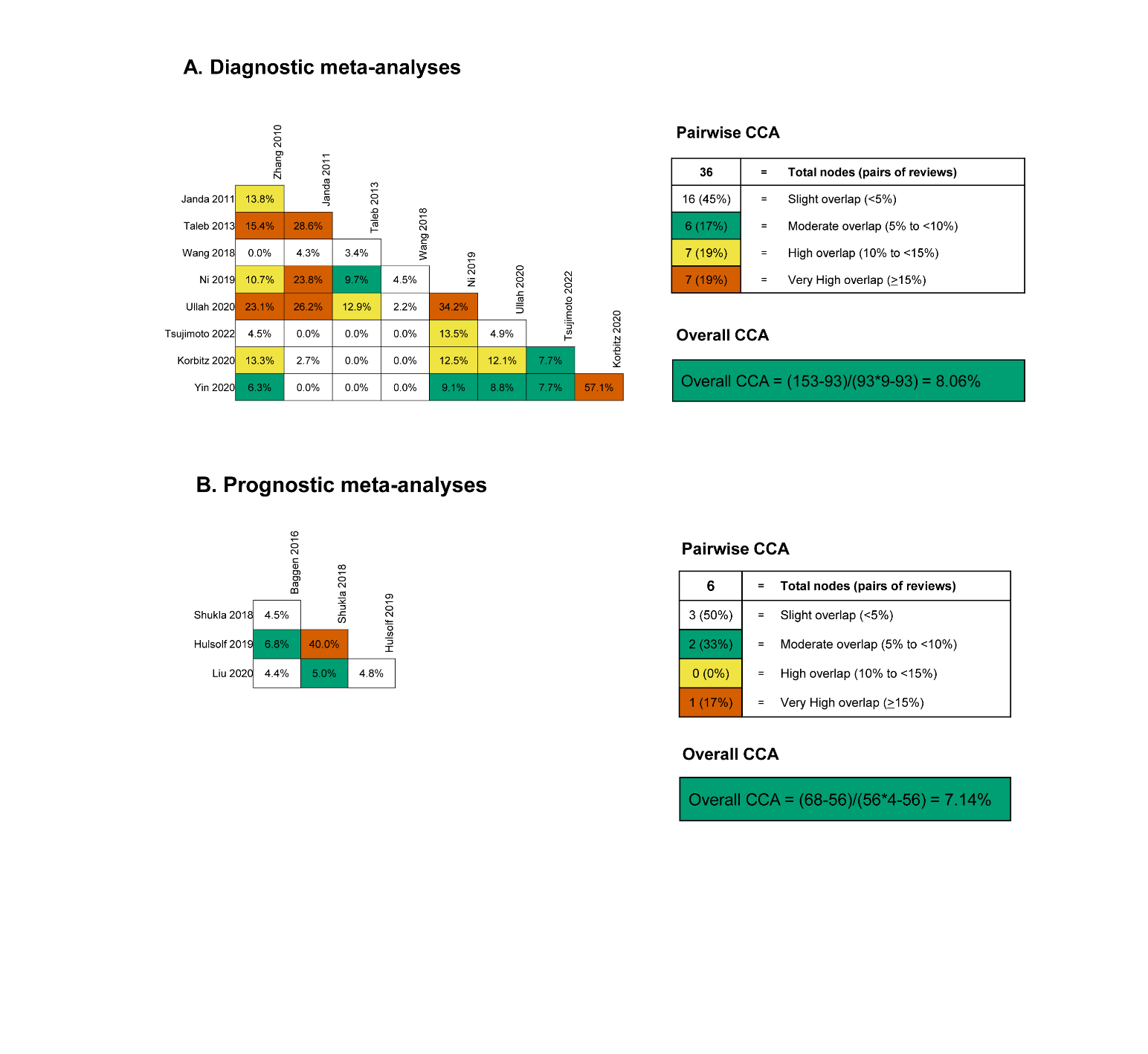


**Supplementary Figure 1.** Overall overlap assessment and pairwise overlap assessment

CCA, corrected covered area

**Availability of data and materials:** All data generated or analyzed in the course of this study are included in these published articles.

1. Zhang RF, Zhou L, Ma GF, Shao FC, Wu XH, Ying KJ. Diagnostic value of transthoracic Doppler echocardiography in pulmonary hypertension: a meta-analysis. American journal of hypertension. 2010;23(12):1261-4.
2. Janda S, Shahidi N, Gin K, Swiston J. Diagnostic accuracy of echocardiography for pulmonary hypertension: a systematic review and meta-analysis. Heart (British Cardiac Society). 2011;97(8):612-22.
3. Taleb M, Khuder S, Tinkel J, Khouri SJ. The diagnostic accuracy of Doppler echocardiography in assessment of pulmonary artery systolic pressure: a meta-analysis. Echocardiography (Mount Kisco, NY). 2013;30(3):258-65.
4. Baggen VJM, Driessen MMP, Post MC, van Dijk AP, Roos-Hesselink JW, van den Bosch AE, et al. Echocardiographic findings associated with mortality ortransplant in patients with pulmonary arterial hypertension:A systematic review and meta-analysis. Netherlands heart journal : monthly journal of the Netherlands Society of Cardiology and the Netherlands Heart Foundation. 2016;24(6):374-89.
5. Shukla M, Park JH, Thomas JD, Delgado V, Bax JJ, Kane GC, et al. Prognostic Value of Right Ventricular Strain Using Speckle-Tracking Echocardiography in Pulmonary Hypertension: A Systematic Review and Meta-analysis. The Canadian journal of cardiology. 2018;34(8):1069-78.
6. Wang YC, Huang CH, Tu YK. Pulmonary Hypertension and Pulmonary Artery Acceleration Time: A Systematic Review and Meta-Analysis. Journal of the American Society of Echocardiography : official publication of the American Society of Echocardiography. 2018;31(2):201-10.e3.
7. Hulshof HG, Eijsvogels TMH, Kleinnibbelink G, van Dijk AP, George KP, Oxborough DL, et al. Prognostic value of right ventricular longitudinal strain in patients with pulmonary hypertension: a systematic review and meta-analysis. European heart journal Cardiovascular Imaging. 2019;20(4):475-84.
8. Ni JR, Yan PJ, Liu SD, Hu Y, Yang KH, Song B, et al. Diagnostic accuracy of transthoracic echocardiography for pulmonary hypertension: a systematic review and meta-analysis. BMJ open. 2019;9(12):e033084.
9. Liu K, Zhang C, Chen B, Li M, Zhang P. Association between right atrial area measured by echocardiography and prognosis among pulmonary arterial hypertension: a systematic review and meta-analysis. BMJ open. 2020;10(9):e031316.
10. Korbitz PM, Gallagher JP, Samant H, Singh S, Jophlin L, Ingviya T, et al. Performance of echocardiography for detection of portopulmonary hypertension among liver transplant candidates: Meta-analysis. Clinical transplantation. 2020;34(11):e13995.
11. Yin X, Shao Y, Zhang Y, Gao H, Qin T, Wen X, et al. Role of echocardiography in screening for portopulmonary hypertension in liver transplant candidates: a meta-analysis. PeerJ. 2020;8:e9243.
12. Ullah W, Minalyan A, Saleem S, Nadeem N, Abdullah HM, Abdalla A, et al. Comparative accuracy of non-invasive imaging versus right heart catheterization for the diagnosis of pulmonary hypertension: A systematic review and meta-analysis. International journal of cardiology Heart & vasculature. 2020;29:100568.
13. Tsujimoto Y, Kumasawa J, Shimizu S, Nakano Y, Kataoka Y, Tsujimoto H, et al. Doppler trans-thoracic echocardiography for detection of pulmonary hypertension in adults. The Cochrane database of systematic reviews. 2022;5(5):Cd012809.

| **Section and Topic** | **Item #** | **Checklist item** | **Location where item is reported** |
| --- | --- | --- | --- |
| **TITLE** | | |  |
| Title | 1 | Identify the report as a systematic review. | 1 |
| **ABSTRACT** | | |  |
| Abstract | 2 | See the PRISMA 2020 for Abstracts checklist. | 1 |
| **INTRODUCTION** | | |  |
| Rationale | 3 | Describe the rationale for the review in the context of existing knowledge. | 2 |
| Objectives | 4 | Provide an explicit statement of the objective(s) or question(s) the review addresses. | 2 |
| **METHODS** | | |  |
| Eligibility criteria | 5 | Specify the inclusion and exclusion criteria for the review and how studies were grouped for the syntheses. | 2 |
| Information sources | 6 | Specify all databases, registers, websites, organisations, reference lists and other sources searched or consulted to identify studies. Specify the date when each source was last searched or consulted. | 2 |
| Search strategy | 7 | Present the full search strategies for all databases, registers and websites, including any filters and limits used. | Additional Files P2 |
| Selection process | 8 | Specify the methods used to decide whether a study met the inclusion criteria of the review, including how many reviewers screened each record and each report retrieved, whether they worked independently, and if applicable, details of automation tools used in the process. | 2 |
| Data collection process | 9 | Specify the methods used to collect data from reports, including how many reviewers collected data from each report, whether they worked independently, any processes for obtaining or confirming data from study investigators, and if applicable, details of automation tools used in the process. | 2 |
| Data items | 10a | List and define all outcomes for which data were sought. Specify whether all results that were compatible with each outcome domain in each study were sought (e.g. for all measures, time points, analyses), and if not, the methods used to decide which results to collect. | 2 |
|  | 10b | List and define all other variables for which data were sought (e.g. participant and intervention characteristics, funding sources). Describe any assumptions made about any missing or unclear information. | 2 |
| Study risk of bias assessment | 11 | Specify the methods used to assess risk of bias in the included studies, including details of the tool(s) used, how many reviewers assessed each study and whether they worked independently, and if applicable, details of automation tools used in the process. | 2 |
| Effect measures | 12 | Specify for each outcome the effect measure(s) (e.g. risk ratio, mean difference) used in the synthesis or presentation of results. | 2, 3, 4 |
| Synthesis methods | 13a | Describe the processes used to decide which studies were eligible for each synthesis (e.g. tabulating the study intervention characteristics and comparing against the planned groups for each synthesis (item #5)). | not required |
|  | 13b | Describe any methods required to prepare the data for presentation or synthesis, such as handling of missing summary statistics, or data conversions. | not required |
|  | 13c | Describe any methods used to tabulate or visually display results of individual studies and syntheses. | not required |
|  | 13d | Describe any methods used to synthesize results and provide a rationale for the choice(s). If meta-analysis was performed, describe the model(s), method(s) to identify the presence and extent of statistical heterogeneity, and software package(s) used. | not required |
|  | 13e | Describe any methods used to explore possible causes of heterogeneity among study results (e.g. subgroup analysis, meta-regression). | not required |
|  | 13f | Describe any sensitivity analyses conducted to assess robustness of the synthesized results. | not required |
| Reporting bias assessment | 14 | Describe any methods used to assess risk of bias due to missing results in a synthesis (arising from reporting biases). | 2 |
| Certainty assessment | 15 | Describe any methods used to assess certainty (or confidence) in the body of evidence for an outcome. | 2, 5 |
| **RESULTS** | | |  |
| Study selection | 16a | Describe the results of the search and selection process, from the number of records identified in the search to the number of studies included in the review, ideally using a flow diagram. | 5 |
|  | 16b | Cite studies that might appear to meet the inclusion criteria, but which were excluded, and explain why they were excluded. | 5 |
| Study characteristics | 17 | Cite each included study and present its characteristics. | 5, 6 |
| Risk of bias in studies | 18 | Present assessments of risk of bias for each included study. | 6 |
| Results of individual studies | 19 | For all outcomes, present, for each study: (a) summary statistics for each group (where appropriate) and (b) an effect estimate and its precision (e.g. confidence/credible interval), ideally using structured tables or plots. | 3, 4, 5, 6 |
| Results of syntheses | 20a | For each synthesis, briefly summarise the characteristics and risk of bias among contributing studies. | not required |
|  | 20b | Present results of all statistical syntheses conducted. If meta-analysis was done, present for each the summary estimate and its precision (e.g. confidence/credible interval) and measures of statistical heterogeneity. If comparing groups, describe the direction of the effect. | not required |
|  | 20c | Present results of all investigations of possible causes of heterogeneity among study results. | not required |
|  | 20d | Present results of all sensitivity analyses conducted to assess the robustness of the synthesized results. | not required |
| Reporting biases | 21 | Present assessments of risk of bias due to missing results (arising from reporting biases) for each synthesis assessed. | 6 |
| Certainty of evidence | 22 | Present assessments of certainty (or confidence) in the body of evidence for each outcome assessed. | 3, 4, 6 |
| **DISCUSSION** | | |  |
| Discussion | 23a | Provide a general interpretation of the results in the context of other evidence. | 6, 7, 8 |
|  | 23b | Discuss any limitations of the evidence included in the review. | 6, 7, 8 |
|  | 23c | Discuss any limitations of the review processes used. | 8 |
|  | 23d | Discuss implications of the results for practice, policy, and future research. | 8, 9 |
| **OTHER INFORMATION** | | |  |
| Registration and protocol | 24a | Provide registration information for the review, including register name and registration number, or state that the review was not registered. | 1, 2 |
|  | 24b | Indicate where the review protocol can be accessed, or state that a protocol was not prepared. | 1, 2 |
|  | 24c | Describe and explain any amendments to information provided at registration or in the protocol. | 2 |
| Support | 25 | Describe sources of financial or non-financial support for the review, and the role of the funders or sponsors in the review. | 9 |
| Competing interests | 26 | Declare any competing interests of review authors. | 9 |
| Availability of data, code and other materials | 27 | Report which of the following are publicly available and where they can be found: template data collection forms; data extracted from included studies; data used for all analyses; analytic code; any other materials used in the review. | 9  Additional Files P5-6 |

Page MJ, McKenzie JE, Bossuyt PM, Boutron I, Hoffmann TC, Mulrow CD, et al. The PRISMA 2020 statement: an updated guideline for reporting systematic reviews. BMJ 2021;372:n71. doi: 10.1136/bmj n71
